# Formononetin: A Pathway to Protect Neurons

**DOI:** 10.3389/fnint.2022.908378

**Published:** 2022-07-13

**Authors:** Xiaoyu Ma, Juejin Wang

**Affiliations:** ^1^The Second Clinical Medical School, Nanjing Medical University, Nanjing, China; ^2^Department of Physiology, Nanjing Medical University, Nanjing, China

**Keywords:** CNS, formononetin, neuroprotecion, signaling pathway, molecular target

## Abstract

Formononetin (FMN) is a phytoestrogen member of the flavonoid family, which has the pharmacological effects of antioxidative, antihypertensive, antitumor, and anti-infective. FMN demonstrates potential in the prevention and treatment of diseases, specifically neurological diseases, such as traumatic brain injury (TBI), spinal cord injury (SCI), ischemic stroke, cerebral ischemia-reperfusion, Alzheimer’s disease, and nerve tumor. Herein, a literature search is conducted to provide information on the signaling pathways of neuroprotection of formononetin based on the neuroprotective study. The significant neuroprotective function of FMN makes it a novel candidate for the development of drugs targeting the central nervous system.

## Introduction

**FIGURE 1:**
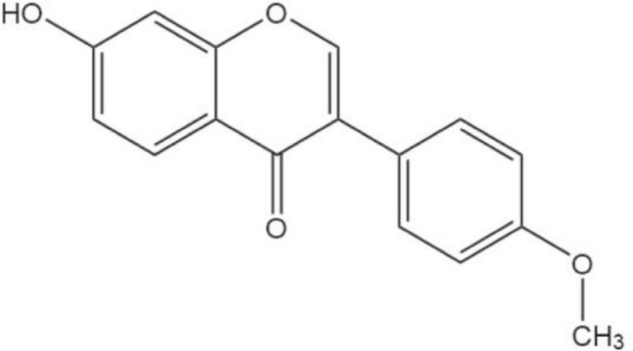
Structure of formononetin.

Formononetin (FMN) is a phytoestrogen member of the flavonoid family, which is the main active component of the legume red clover and one of the active components of commonly used Chinese herbal medicines such as *Angelica sinensis, Astragalus membranaceus, Kudzu root*, and *Caulis spatholobi*. The chemical molecular formula of FMN (C_16_H_12_O_4_) is shown in Figure 1. FMN can be obtained from natural plants by drug extraction and purification methods, such as extraction, ultrasound, microwave, supercritical water application, and pressurization, and can also be synthesized and modified by artificial methods. FMN has antioxidative, antihypertensive, antitumor, anti-infective, estrogen-like, and other pharmacological effects (Machado Dutra et al., 2021).

In the past few decades, FMN has been intensively investigated in the repair of tissues, following lung injury (Chen et al., 2021), kidney injury (Huang et al., 2017; Oza and Kulkarni, 2019; Lv et al., 2020), myocardial injury (Wang et al., 2020), atherosclerosis (Sun et al., 2013; Ma et al., 2020), peripheral nerve injury (Fang et al., 2020; Oza and Kulkarni, 2020), wound healing (Li et al., 2015), articular cartilage metabolism (Cho et al., 2019), and hair regeneration (Kim et al., 2016; El-Arabey and Abdalla, 2020). It is actively involved in antibacterial and antifungal activities against candida strains (das Neves et al., 2016), and also provides inhibitory effects on various malignant tumors, such as gastric cancer (Yao et al., 2019; Wang and Zhao, 2021), bladder cancer (Wu et al., 2017), and breast cancer (Zhou et al., 2019). In addition, FMN demonstrates potential in the prevention and treatment of diseases, specifically neurological diseases, such as Alzheimer’s disease (AD) and cerebral ischemia (Sun et al., 2011). Thus, we aim to introduce the central neuroprotective role and mechanism of FMN by a mini review.

## Traumatic Brain Injury

**FIGURE 2:**
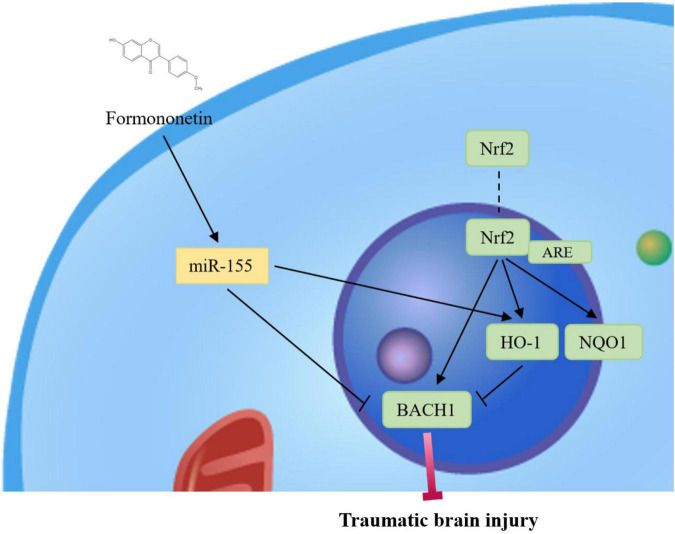
The underlying mechanisms of FMN involved in TBI.

Traumatic brain injury (TBI) is a brain injury caused by external force, and oxidative stress may be one of the main causes of TBI. Interestingly, growing evidence suggests that FMN has antioxidant properties. Therefore, FMN may have potential applications in the treatment of oxidative stress injury in TBI. Specifically, FMN may protect TBI rats from neurological damage, and the mechanism underlying this protection is related to the inhibition of intracranial inflammatory responses and oxidative stress (Li et al., 2014). Recently, FMN has been found to upregulate the expression of nuclear factor E2-related factor 2 (Nrf2) in TBI rats (Berry et al., 2009; Soltani et al., 2015; Engler-Chiurazzi et al., 2017). Li et al. (2017) found that FMN, a typical isoflavone phytoestrogen, improves brain dysfunction, reduces brain edema, and inhibits neuronal apoptosis. Moreover, the administration of FMN attenuated TBI-induced oxidative stress by upregulating heme oxygenase-1 (HO-1) expression and downregulating the BTB domain and CNC homolog 1 expression. This process increases the expression of miR-155, which suggests that FMN exerts neuroprotective effects by regulating key molecules related to the Nrf2/antioxidant response element [ARE]/HO-1 antioxidant pathway and regulating redox homeostasis in TBI rats. Accumulating evidence presents that the Nrf2/ARE/HO-1 pathway is involved in oxidative stress in secondary brain injury, following TBI (Figure 2; Cheng et al., 2016; Liu Z. et al., 2017). As a key regulator of ARE, Nrf2 activates transcription in response to oxidative stress; however, this process is significantly different between animal models and clinical treatments. To verify the relationship between the Nrf2/ARE/HO-1 pathway activation and FMN efficacy in the treatment of TBI, further studies should evaluate the mechanism of injury response and functional deficits (Figure 2).

In addition, Li et al. (2014) suggested that TBI increases the levels of tumor necrosis factor-α (TNF-α) and interleukin (IL)-6 and upregulates cyclooxygenase-2 (COX-2) mRNA levels in damaged brain areas. The results are consistent with significant hydrocephalus in rats model. Therefore, the inflammatory response is believed to accelerate TBI deterioration. Following FMN treatment, a significant decrease was noted in the concentration of inflammatory cytokines in the damaged brain, accompanied by a corresponding decrease in COX-2 mRNA expression. The inhibition of COX-2 activity in astrocytes, neurons, and microglia by FMN effectively reduces intracranial inflammatory mediators, such as TNF-α and IL-6. The modulation of the inflammatory cascade in the central nervous system may be a therapeutic target for TBI. TBI results in significant cell necrosis or death in the cortex, which induces nerve injury (Li et al., 2018). In biological mechanisms, FMN-mediated activation of IL-10 expression promotes cell growth by binding to IL-10-specific receptors in cortical neurons, which subsequently inhibits cortical injury in TBI. Increased serum IL-10 levels with FMN treatment may be related to IL-10-based biological pathways in peripheral cortical neurons to counteract TBI conditions. These studies have demonstrated that FMN may have neuroprotective effects against TBI by inducing neuronal IL-10 expression. Collectively, FMN is implicated in suppressing inflammatory responses and oxidative stress in the brain to enhance neuroprotection, and, thus, FMN may be promising agent for the treatment of TBI.

## Spinal Cord Injury

Spinal cord injury (SCI) has become a challenging chronic problem because of the interaction of multiple factors such as ischemia, oxidative stress, excitotoxicity, and immune-inflammatory response in the damaged local tissues. SCI results in neuronal and oligodendrocyte apoptosis and necrosis, peripheral syrinx, and glial scar formation in the damaged area, and limited regenerative capacity of the central nervous system. However, few effective strategies for promoting nerve regeneration in SCI have been established. Thus, the use of growth-permissive substrates is necessary at the injury site (Roman et al., 2011; Yamane et al., 2018).

Given that gelatin methacryloyl (GelMA) has excellent biocompatibility and mechanical properties, favoring cell adhesion and proliferation, it can be used for nerve tissue repair at diseased sites (Soucy et al., 2018). de Vasconcelos et al. (2020) used FMN to assess the functionalization process of carbon nanotubes (CNs) and developed a GelMA formulation, containing CN-FMN that was applied to spinal cord lesions by *in situ* photopolymerization. CN has been evaluated for the treatment of SCI because of its electrical properties and nanometer size. A photopolymerized preparation containing FMN-functionalized CNs was developed for *in situ* SCI treatment; thus, functionalized CNs can be incorporated into GelMA formulations. Constant hydrogel formation *in situ* after exposure to UV radiation makes this product a treatment option for SCI.

In addition, Novais et al. (2021) further developed a GelMA-hydroalcoholic extract of red propolis (HERP) formulation (the main bioactive maker of the formulation is FMN), and verified the function of the formulation in the experimental model of rat SCI. The results showed that the formulations containing FMN had greater post-injury recovery rates compared to formulations without FMN. Histomorphometric techniques demonstrated that the GelMA-HERP formulations treated tissue with less inflammation and less cavitation at the injury site.

## Ischemic Stroke

In animal models of local and global cerebral ischemia, the dietary intake of phytoestrogens may reduce stroke injury (Lovekamp-Swan et al., 2007; Liang et al., 2008; Donzelli et al., 2010). However, the potential protective effects of phytoestrogens on ischemic injury are still unclear (Schreihofer and Redmond, 2009). Recent studies have shown that FMN has neuroprotective effects on ischemic stroke through its anti-apoptotic effects (Liang et al., 2014). FMN may protect against ischemic stroke by downregulating the Bax/Bcl-2 ratio, activating PI3K/AKT and anti-apoptosis, and reducing the TNF-α level (Li et al., 2014; Liang et al., 2014). However, whether FMN can restore neurological functions after ischemic stroke is still unknown.

Axons are components of neurons, and axonal growth promotes the differentiation of damaged neurons, establishes new connections with other neurons, forms neural networks, and restores impaired neurological function (Rao and Pearse, 2016). βIII-tubulin is a protein that makes up the neuronal skeleton, and the upregulation of βIII-tubulin expression can modify the axonal cytoskeleton, which is important for the regrowth of axons (Moskowitz and Oblinger, 1995). Growth-associated protein 43 (GAP-43) is a neuron-specific axonal membrane protein involved in the regulation of the growth and synaptic development of neurons (Donnelly et al., 2013). FMN upregulated the expressions of βIII-tubulin and GAP-43, indicating that it may induce neuronal differentiation. The nerve growth factor (NGF) is involved in the development, differentiation, growth, regeneration, and functional properties of neurons (Aloe et al., 2015). Synaptic connections between nerve cells are called synaptic plasticity and are the basis for neuronal regeneration and repair (Castrén and Antila, 2017). Brain-derived neurotrophic factor (BDNF) is typically involved in synaptic plasticity and promotes neuronal development, differentiation, growth, and regeneration (Begni et al., 2017). Wu et al. (2020) found FMN significantly increases expressions of NGF and BDNF, which may contribute to the beneficial effects of FMN on synaptic plasticity to restore neurological function.

It is worth noting that, although there are gender differences in stroke-induced injury and stroke repair, most of the current animal models are based on male rats, so future studies should further explore the gender differences in FMN treatment of stroke.

## Cerebral Ischemia Reperfusion

**FIGURE 3:**
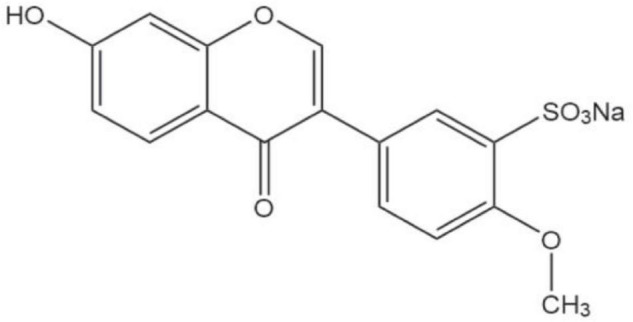
Structure of SUL-F.

Cerebral ischemia reperfusion (I/R) injury leads to a series of injuries, including cell death (necrosis and apoptosis), brain edema, and other cellular reactions, such as angiogenesis and reconstruction of functional microvessels to promote stroke recovery (Folkman, 1995; Beck and Plate, 2009; Chen et al., 2012). Vascular endothelial growth factor (VEGF)-signaling proteins are stimulated by ischemia and are essential for the angiogenesis and prevention of ischemic injury (Carmeliet, 2003). The upregulation of VEGF not only promotes angiogenesis but also increases microvascular permeability (Dvorak et al., 1995). Platelet endothelial cell adhesion molecule 1 (PECAM-1) is a member of the immunoglobulin gene superfamily of cell adhesion molecules and is composed of all contiguous human endothelial cells (Muller et al., 1992). PECAM-1 mediates cell-cell adhesion through homophilic and heterophilic interactions and transduces intracellular signals that upregulate integrin function on leukocytes (Delisser et al., 1997). At present, sodiumformononetin-3′-sulfonate (SUL-F) has been synthesized. SUL-F (3, 7.5, 15, and 30 mg/kg administered intravenously) was found to have protective effects against cerebral I/R injury by improving neurological function, inhibiting apoptosis, and increasing the expression levels of VEGF and PECAM-1 caused by middle cerebral artery occlusion (Figure 3). Treatment with SUL-F (10 and 20 μg/ml) significantly increased cell migration, angiogenesis, and VEGF and PECAM levels in human umbilical vein endothelial cells (Zhu et al., 2014). SUL-F was found to have significant neuroprotective effects on cerebral I/R injury in rats and could improve cerebral angiogenesis in human umbilical vein endothelial cells. The protective mechanism of SUL-F is attributed to the promotion of the expressions of VEGF and PECAM, inhibition of apoptosis, and improvement of cerebral angiogenesis.

**FIGURE 4:**
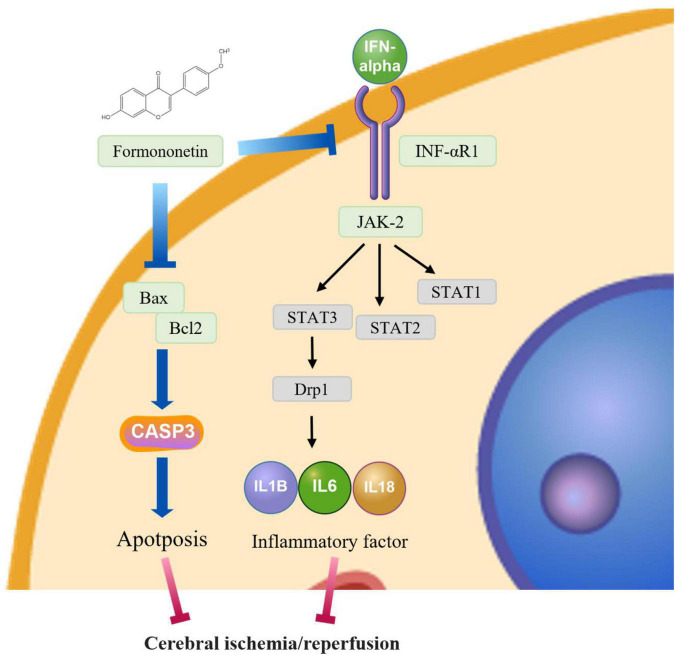
The underlying mechanisms of FMN involved in cerebral ischemia/reperfusion.

Estrogen has neuroprotective properties, and dietary intake of phytoestrogens can attenuate stroke injury in animal I/R models. To investigate the molecular mechanism underlying the neuroprotective effects of FMN on I/R rats, some studies have found that FMN significantly reduces the infarct volume and brain water content, improves neurological deficits, upregulates ER-α and p-Akt, and downregulates the Bax/Bcl-2 ratio, while FMN had little effects on p-ERK1/2 protein expressions (Figure 4; Liang et al., 2014). FMN demonstrated neuroprotective effects on cerebral I/R injury rats, and the molecular mechanism may be associated with the downregulation of the Bax/Bcl-2 ratio and the activation of the PI3K/Akt signaling pathway. FMN has some protective effects on I/R injury rats, and the mechanism may be related to anti-oxidative stress and anti-apoptosis. These results suggest the potential of FMN as an effective and promising agent for the treatment of cerebral I/R injury.

## Alzheimer’s Disease

**FIGURE 5:**
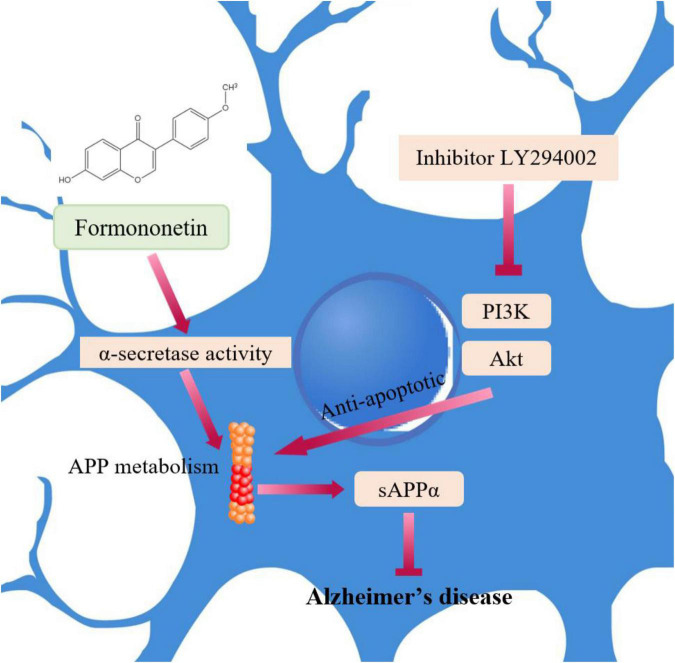
The underlying mechanisms of FMN involved in AD.

Alzheimer’s disease (AD) is the most common cause of dementia in the elderly population. Its main pathological features include the accumulation of extracellular amyloid-β (Aβ) plaques and intracellular neurofibrillary tangles (Bloom, 2014). Studies have established a strong correlation between AD symptoms and the accumulation of these plaques and tangles because they induce neurodegeneration that mediates memory loss and cognitive loss. Disruptions in the Aβ transport across the blood-brain barrier are key elements in the pathogenesis of AD. In the vascular endothelial cells of the hippocampus, Aβ transport is mainly mediated by low-density lipoprotein-associated protein 1 (LRP1) and advanced glycation end receptor (RAGE) products. Fei et al. (2018) investigated the effects of FMN on improving learning and memory in amyloid precursor protein (APP)/presenilin-1 mice and related mechanisms. They found that FMN significantly improved learning and memory by inhibiting APP processing to produce Aβ, RAGE-dependent inflammatory signaling, and promoting LRP1-dependent brain Aβ clearance pathways (Fei et al., 2018). Moreover, FMN treatment alleviated ultrastructural changes in hippocampal vascular endothelial cells. Chen et al. (2017) reported the protective effects of FMN on Aβ25-35-induced neurotoxicity in HT22 cells. FMN significantly increased the viability of HT22 cells and decreased the apoptosis rate when challenged with Aβ25-35 (Chen et al., 2017). The inhibitory effects of FMN were associated with the PI3K/Akt signaling pathway, as the PI3K inhibitor (LY294002) or the ERα-specific inhibitor prevented this effect (Figure 5). The form also accelerates the non-amyloidogenic process of amyloid protein (APP) by enhancing α-secretase activity and sAPPα release (Figure 5). Taken together, FMN may be an effective and promising treatment of AD.

Emerging evidence continues to demonstrate that neuroinflammation is also a major pathological component of AD (Webers et al., 2020). Several animal experimental and clinical studies have also provided a close link between neuroinflammation and AD pathogenesis. Accumulating evidence suggests that the inflammatory response in the brain is a major factor in AD pathogenesis (Heppner et al., 2015; Fu et al., 2019). High levels of proinflammatory mediators were detected in the brains of patients with AD (Hesse et al., 2016). Therefore, the anti-inflammatory effects of FMN have the potential to delay the disease onset or slow disease progression in AD. Diet-based FMN treatment can be applied for neuroprotection against oxidative stress and may reduce the incidence of neurodegenerative diseases. Future studies should investigate the activity of FMN *in vivo* and elucidate more details of the underlying mechanisms.

## Nerve Tumor

For more than a decade, FMN has been intensively investigated because of its apoptosis-promoting and antiproliferative effects and potential as an anticancer agent. These anticancer properties have been observed in various cancer models, such as gastric cancer (Yao et al., 2019; Wang and Zhao, 2021), bladder cancer (Wu et al., 2017), and breast cancer (Zhou et al., 2019). In addition, FMN may also attenuate metastasis and tumor growth. Specifically, FMN may contribute to antiproliferative characteristics and cell cycle arrest-inducing properties of cells (Ong et al., 2019).

In recent years, increasing evidence has shown that most anticancer drugs are derived from natural products, such as including paclitaxel, vinblastine, and camptothecin (Sethi et al., 2012; Cragg and Pezzuto, 2016; Liu D. et al., 2017). For instance, polysaccharides obtained from Astragalus plants can counteract the adverse effects of chemotherapeutic drugs by significantly reducing myelosuppression in the patients with cancer (Duan and Wang, 2002). In addition to its antiproliferative and pro-apoptotic properties, FMN enhances the transcriptional activity of p53 by increasing its phosphorylation at Ser15 and Ser20, thereby upregulating the expression level of p53 in a concentration-dependent manner (Yang et al., 2014). For example, FMN exhibits synergy when combined with other chemotherapeutic agents. Temozolomide (TMZ) is an oral chemotherapeutic agent usually used in the treatment of certain brain cancers, such as glioblastoma multiforme. However, TMZ has undesirable side effects, including hematological complications and intrinsic and acquired resistance (van den Bent et al., 2003; Zhang et al., 2018). While FMN and TMZ alone can sufficiently inhibit the growth of C6 glioma cells in a concentration-dependent manner, they demonstrate a synergistic effect on C6 cells when they are used in combination. This drug combination increased Bax protein expressions, cleaved caspase-3 and caspase-9, attenuated Bcl-2 expression, and promoted tumor cell apoptosis (Zhang et al., 2018). As FMN has protective effects against certain malignancies, FMN has been clinically used as one of the basic herbs for the treatment of cancer in traditional medicine (Wang et al., 2018).

In conclusion, the beneficial effects of FMN can be attributed to its antiproliferative and cell cycle arrest-inducing properties. FMN regulates various transcription factors and growth factor-mediated oncogenic pathways, thereby mitigating possible causes of chronic inflammation associated with the survival of tumor cells and their resistance to chemotherapy.

## Outlook

The evidence presented herein provides a comprehensive summary of the potential neuroprotective effects of FMN in *in vitro* and *in vivo* studies and the current progress in clinical research. Many molecular targets and mechanisms of action are involved in the protective properties of central nervous system diseases. Moreover, numerous *in vitro* studies have demonstrated that the safety and the efficacy of FMN and its metabolites in biological systems are further confirmed in *in vivo* studies. The potential role of FMN in prospective drug development is supported by these findings. However, further *in vivo* and clinical studies should assess the efficacy and safety of FMN in the prevention and treatment of various central nerve conditions. FMN derivatives and metabolites should have different kinetic properties and activities and be fully elucidated; thus, further studies should assess if this bioactive phytochemical is safe for clinical usage. The significant neuroprotective function of FMN makes it a novel candidate for the development of drugs targeting the central nervous system.

## Author Contributions

JW: study concept and design. XM and JW: drafting of the manuscript. Both authors contributed to the article and approved the submitted version.

## Conflict of Interest

The authors declare that the research was conducted in the absence of any commercial or financial relationships that could be construed as a potential conflict of interest.

## Publisher’s Note

All claims expressed in this article are solely those of the authors and do not necessarily represent those of their affiliated organizations, or those of the publisher, the editors and the reviewers. Any product that may be evaluated in this article, or claim that may be made by its manufacturer, is not guaranteed or endorsed by the publisher.
